# DNA damage response as a driver of PD-L1 expression in cellular senescence: implications for immune evasion and immunosenescence

**DOI:** 10.3389/fcell.2026.1974523

**Published:** 2026-10-16

**Authors:** Yajun Wang, Ling Qin

**Affiliations:** 1 School of Medical Technology and Engineering, Henan University of Science and Technology, Luoyang, China; 2 The First Affiliated Hospital, and College of Clinical Medicine of Henan, University of Science and Technology, Luoyang, China

**Keywords:** cellular senescence, DNA damage response, immune evasion, immunosenescence, PD-L1, telomere dysfunction

## Abstract

Aging is accompanied by the progressive accumulation of senescent cells, which contribute to tissue dysfunction and chronic inflammation. These cells persist despite a pro-inflammatory profile that should, in principle, recruit immune cells for their clearance. We propose that this persistence is explained, at least in part, by PD-L1-mediated immune evasion driven by the DNA damage response (DDR). The aim of this Mini Review is to synthesize evidence linking DNA damage response signaling, initiated by diverse senescence-associated stressors, to PD-L1 upregulation and immune evasion in senescence. We discuss how DDR signaling drives PD-L1 expression through transcriptional mechanisms, namely, the cGAS-STING pathway and the ATM/ATR-STAT1/IRF1 axis, and through post-translational stabilization via p16-mediated inhibition of CDK4/6. This review also discusses the function of PD-L1 in the nucleus, where it interacts with cohesin-SA1 to maintain telomere cohesion. Finally, it highlights that DDR-driven PD-L1 upregulation establishes a self-reinforcing circuit: immune evasion allows senescent cells to accumulate, which exacerbates telomere dysfunction in neighboring cells and reinforces the senescent phenotype. This framework recasts senescence as an actively maintained immunoevasive condition and identifies the DDR-PD-L1 axis as a potential target for restoring immune clearance of senescent cells.

## Introduction

Cellular senescence arrests cell cycle progression in response to diverse stressors, including telomere attrition, DNA damage, oncogene activation, and mitochondrial dysfunction. When such cells persist in aging tissues, they acquire a senescence associated secretory phenotype (SASP) that promotes chronic inflammation, tissue dysfunction, and age-related pathology (Herranz and Gil, 2018; Muñoz-Espín and Serrano, 2014; Campisi, 2013). This raises a key question. If senescent cells display a pro-inflammatory profile that should recruit immune cells for clearance, why do they instead evade elimination and accumulate with age?

The PD-1/PD-L1 immune checkpoint is known for its role in tumor immune evasion, and recent evidence indicates that it operates similarly in senescence. Senescent cells upregulate PD-L1, engage PD-1 on cytotoxic T cells and natural killer cells, and thereby dampen their own immune clearance (Salminen, 2024; Keir et al., 2008; Majewska et al., 2024). This offers a partial explanation for the question above. But the explanation is incomplete, as the upstream signals that drive PD-L1 upregulation in senescent cells have not been fully defined.

We propose that the DNA damage response (DDR), a conserved signaling network rapidly activated by genotoxic stress, acts as a key upstream driver of PD-L1 expression in senescence through both transcriptional and post-translational mechanisms, thereby linking senescence-associated genomic instability to immune checkpoint activation. The DDR can be engaged by multiple senescence-associated stressors, including telomere dysfunction, oxidative damage, and replication stress. Among these, telomere attrition has been the most extensively studied in the context of replicative senescence (Shay and Wright, 2000) and will be a central focus of this review. DDR signaling drives PD-L1 expression through two distinct routes: transcriptional induction via the cGAS-STING pathway and post-translational stabilization through p16-mediated inhibition of CDK4/6. In addition, a portion of PD-L1 localizes to the nucleus, where it interacts with cohesin SA1 to promote telomere cohesion, extending PD-L1’s known functions beyond immune regulation. The DDR–PD-L1 axis thus forms a self-reinforcing circuit that perpetuates senescent cell accumulation and immunosenescence. This framework explains how senescent cells persist in aging tissues and points to potential therapeutic nodes for restoring immune mediated clearance.

## Diverse stressors engage the DDR in senescent cells

DDR is not triggered by a single type of insult. In senescent cells, the DDR can be activated by multiple stressors, including telomere dysfunction, oxidative damage, and replication stress. Among these, telomere dysfunction has been studied most extensively. Telomeres, the protective caps at chromosome ends, shorten with each round of DNA replication in the absence of sufficient telomerase activity. When telomeres become critically short, they lose their protective structure and are recognized by the DNA repair machinery as double-strand breaks (Palm and de Lange, 2008; de Lange, 2018; Casari et al., 2022). This recognition initiates a DDR that becomes persistent in replicative senescence, distinguishing it from transient cell cycle arrest (d'Adda di Fagagna et al., 2003; d'Adda di Fagagna, 2008).

But telomeres are not the only source. Oxidative stress, which accumulates with age, generates reactive oxygen species (ROS) that damage nuclear DNA and activate DDR signaling (Ksiazek et al., 2007). Replication stress, mainly arising from conflicts between DNA replication and transcription or from insufficient replication origins, also engages the DDR in senescent cells (Zeman and Cimprich, 2014; Vanzo et al., 2020). In each of these scenarios, the DDR can persist and shape cellular behavior beyond the immediate DNA repair response, including, as we discuss below, the regulation of immune checkpoint molecules (PD-L1). The DDR–PD-L1 axis is not restricted to replicative senescence but is likely engaged across a broader range of senescence contexts.

## DDR–PD-L1 upregulation mechanisms

DDR signaling drives PD-L1 expression through both transcriptional and post-translational mechanisms. The transcriptional route is well characterized through the cGAS-STING pathway. DNA damage can generate cytosolic DNA, including micronuclei arising from genomic instability. Upon micronuclear envelope rupture, this DNA is sensed by cGAS and can trigger STING activation. STING then recruits TBK1, which phosphorylates IRF3, leading to type I interferon production and subsequent PD-L1 transcription (Sun et al., 2013; Li and Chen, 2018). In senescent cells, this same cascade is engaged by cytoplasmic chromatin fragments (Glück et al., 2017; Yang et al., 2017). Beyond the cGAS–STING axis, ATM and ATR signaling also contribute to PD-L1 regulation. ATM (ataxia-telangiectasia mutated) and ATR (ATM- and Rad3-related) are central kinases that initiate DNA damage response signaling. Following DNA damage, the ATM/ATR/Chk1 kinase cascade activates STAT1/STAT3 signaling and induces IRF1 expression, both of which drive PD-L1 transcription. ATM can also enhance PD-L1 expression through NF-κB activation (Sato et al., 2017). Figure 1 summarizes the proposed pathway linking DDR signaling, ATM/ATR activation, cGAS-STING pathway, PD-L1 regulation, and immune evasion. In addition to these pathways, the role of p53, a central DDR effector, deserves mention, as its impact on PD-L1 expression is context dependent and may vary in senescent cells.

**FIGURE 1:**
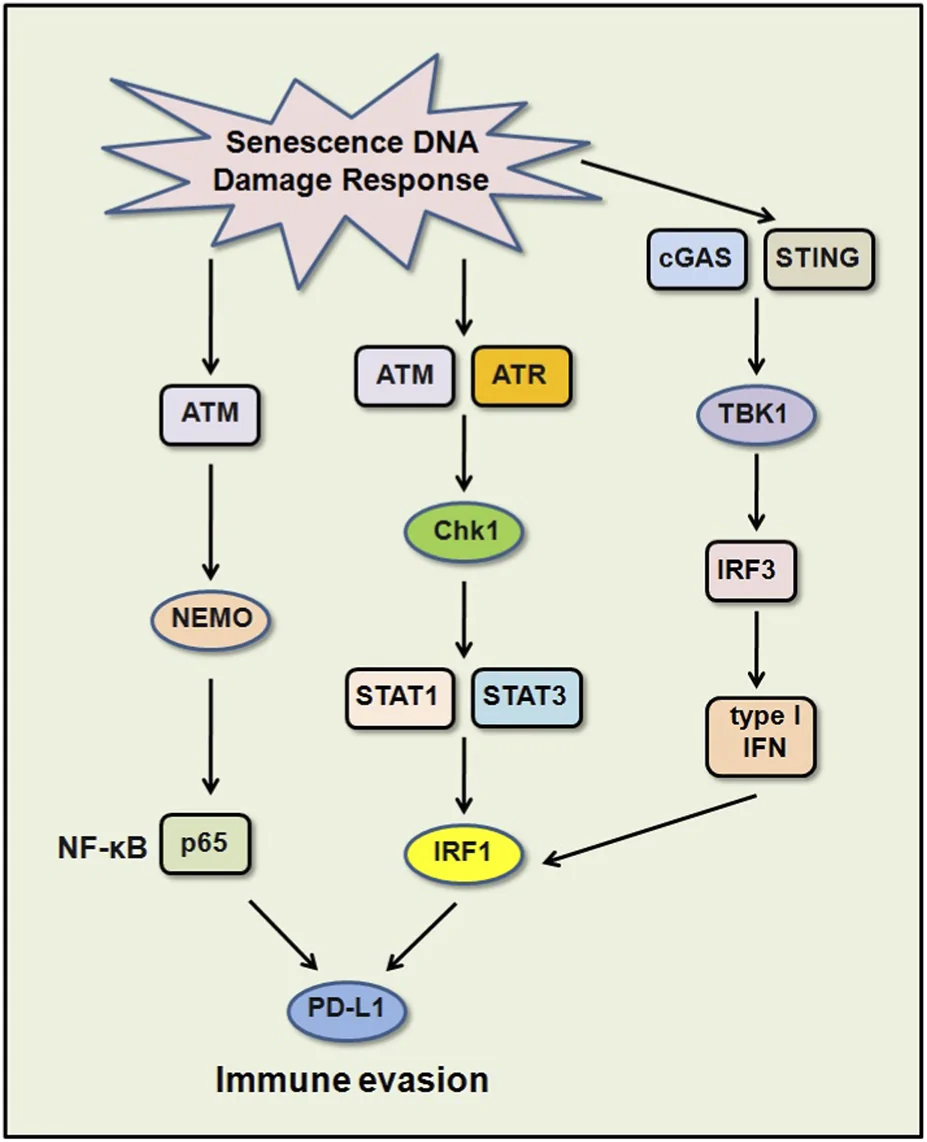
Mechanisms linking DDR signaling to PD-L1 expression and immune evasion in senescence. Senescence-associated stressors activate the DNA damage response (DDR). ATM and ATR are activated by distinct DNA lesions and trigger downstream signaling. ATM/ATR/Chk1 kinase signaling activates STAT1/STAT3, which induces IRF1 expression; IRF1 then binds the PD-L1 promoter and promotes transcription. In parallel, ATM can activate NF-κB through NEMO, leading to p65 nuclear translocation and PD-L1 transcription. The cGAS-STING pathway also promotes PD-L1 transcription through TBK1-IRF3 and type I interferon-JAK/STAT signaling. Elevated surface PD-L1 engages PD-1 on cytotoxic T cells and natural killer (NK) cells, enabling immune evasion and senescent cell accumulation. Solid arrows indicate established causal relationships.

But transcriptional upregulation is not the only mechanism. A post-translational route also reinforces PD-L1 accumulation in senescence: p16-mediated inhibition of CDK4/6 limits ubiquitin-dependent proteasomal degradation of PD-L1, thereby stabilizing the protein independently of transcriptional changes (Majewska et al., 2024). This pathway may sustain PD-L1 expression even when transcriptional input declines. DDR-driven transcription and p16-mediated stabilization thus cooperate to maintain PD-L1 expression in senescence. This dual control may render senescent cells inherently difficult for the immune system to clear. A summary of the key mechanisms discussed in this section is provided in Table 1.

**TABLE 1:** Mechanisms linking DDR signaling to PD-L1 regulation.

Regulatory layer	Pathway	Key mediators	Effect on PD-L1	References
Transcriptional	cGAS-STING	cGAS, STING, TBK1, IRF3, type I IFN, JAK/STAT, IRF1	Promotes transcription	Sun et al. (2013); Li and Chen (2018); Glück et al. (2017); Yang et al. (2017)
Transcriptional	ATM/ATR	ATM, ATR, Chk1, STAT1/STAT3, IRF1, NF-κB	Promotes transcription	Sato et al. (2017)
Post-translational	p16-CDK4/6	p16, CDK4/6, ubiquitin-proteasome	Stabilizes protein	Majewska et al. (2024)

DDR, DNA damage response.

## PD-L1 enables senescent cell immune evasion

PD-L1 exerts its immunosuppressive effects by binding to PD-1 on cytotoxic immune cells (Keir et al., 2008; Sharpe and Pauken, 2018). Upon engagement, PD-1 recruits SHP-1 and SHP-2 phosphatases, which dephosphorylate key components of the T cell receptor signaling cascade. This dampens T cell activation, proliferation, and cytokine production (Boussiotis, 2016; Rabinovich et al., 2007). The same pathway also limits NK cell cytotoxic function (Giuliani et al., 2017).

In the context of senescence, p16-positive alveolar macrophages in aged lungs upregulate PD-L1 through p16-mediated inhibition of CDK4/6. This inhibition limits ubiquitin-dependent degradation of PD-L1 and stabilizes the protein (Majewska et al., 2024). These PD-L1-high senescent macrophages create an immunosuppressive microenvironment that favors senescent cell accumulation. Single-cell analyses have revealed a correlation between p16 and PD-L1 expression, seen in both immune and epithelial cells, and more pronounced in older mice. Senescent cells that display enhanced MHC class I machinery also express PD-L1, suggesting that they can present antigens while simultaneously using PD-L1 to engage PD-1 on T cells, potentially driving T cell exhaustion (Zhou et al., 2025).

*In vivo* studies provide functional validation of this axis. Activating anti-PD-L1 antibodies that engage Fcγ receptors on effector cells eliminate PD-L1 positive senescent cells and reduce senescence-associated inflammation, both in naturally aged mice and in models of chronic lung inflammation (Majewska et al., 2024). These data indicate that PD-L1 is not merely a senescence marker but a functionally relevant checkpoint that protects senescent cells from immune elimination.

## The two faces of PD-L1 in senescent cells: immune evasion and genomic protection

The preceding sections have established that senescent cells upregulate PD-L1 at the cell surface to evade immune clearance. But recent work has uncovered a second function, one that operates not at the surface, but in the nucleus. A fraction of PD-L1 translocates to the nucleus through an importin-dependent pathway involving KPNA2 and KPNA6. Once inside, it binds directly to the cohesin subunit SA1 (STAG1) via its cytoplasmic tail. And this interaction is required for SA1 nuclear localization and proper telomere maintenance. Depletion of either PD-L1 or SA1 produces comparable genomic instability phenotypes, aneuploidy, telomere cohesion defects, and reduced proliferation. And double knockdown shows no synergistic effect, suggesting they act in the same pathway (Zhang et al., 2021).

This raises a question about the logic of this feedback. In senescent cells with eroded telomeres, PD-L1 upregulation serves two purposes: immune evasion at the surface and telomere protection in the nucleus. Both outcomes help the cell survive. What makes this interesting is the circularity: the same telomere dysfunction that triggers DDR and PD-L1 upregulation is also mitigated by PD-L1’s nuclear function. The response is mounted after the damage has occurred, and it feeds back to limit further damage.

The temporal dynamics of senescence help to make sense of this feedback loop. Telomere attrition triggers DDR-driven PD-L1 upregulation; once expressed, a fraction of PD-L1 translocates to the nucleus and helps preserve the integrity of the remaining telomeres. The response is therefore mounted after the dysfunction has occurred, and serves to limit further damage rather than to prevent the initial attrition. This is not a paradox, it is damage control. At the mechanistic level, PD-L1 maintains genomic integrity while simultaneously enabling immune evasion, allowing a potentially harmful cell to persist. This nuclear function thus adds a layer of complexity to PD-L1’s role in senescence, beyond its familiar function as an immune checkpoint.

## The self-reinforcing circuit

We have described how DDR signaling drives PD-L1 upregulation, how PD-L1 enables immune evasion, and how a fraction of PD-L1 also functions in the nucleus to maintain telomere cohesion. These observations point to a circuit (Figure 2). Telomere attrition and other senescence-associated stressors engage a persistent DDR (d'Adda di Fagagna et al., 2003; Di Micco et al., 2021). This DDR upregulates PD-L1 through transcriptional induction and post-translational stabilization (Li and Chen, 2018; Majewska et al., 2024). Elevated PD-L1 then allows senescent cells to evade immune clearance and accumulate (Herranz and Gil, 2018; Campisi, 2013). These cells secrete SASP factors that promote chronic inflammation (Muñoz Espín and Serrano, 2014; Franceschi and Campisi, 2014). The inflammatory milieu, in turn, induces DNA damage and telomere dysfunction in neighboring cells (Lagnado et al., 2021; Victorelli and Passos, 2020), feeding back to the start. This is mediated in part by SASP-driven recruitment of neutrophils, which transfer ROS to neighboring cells and inflict telomeric damage. Each round amplifies the burden of senescent cells (Sharma, 2024; Childs et al., 2015).

**FIGURE 2:**
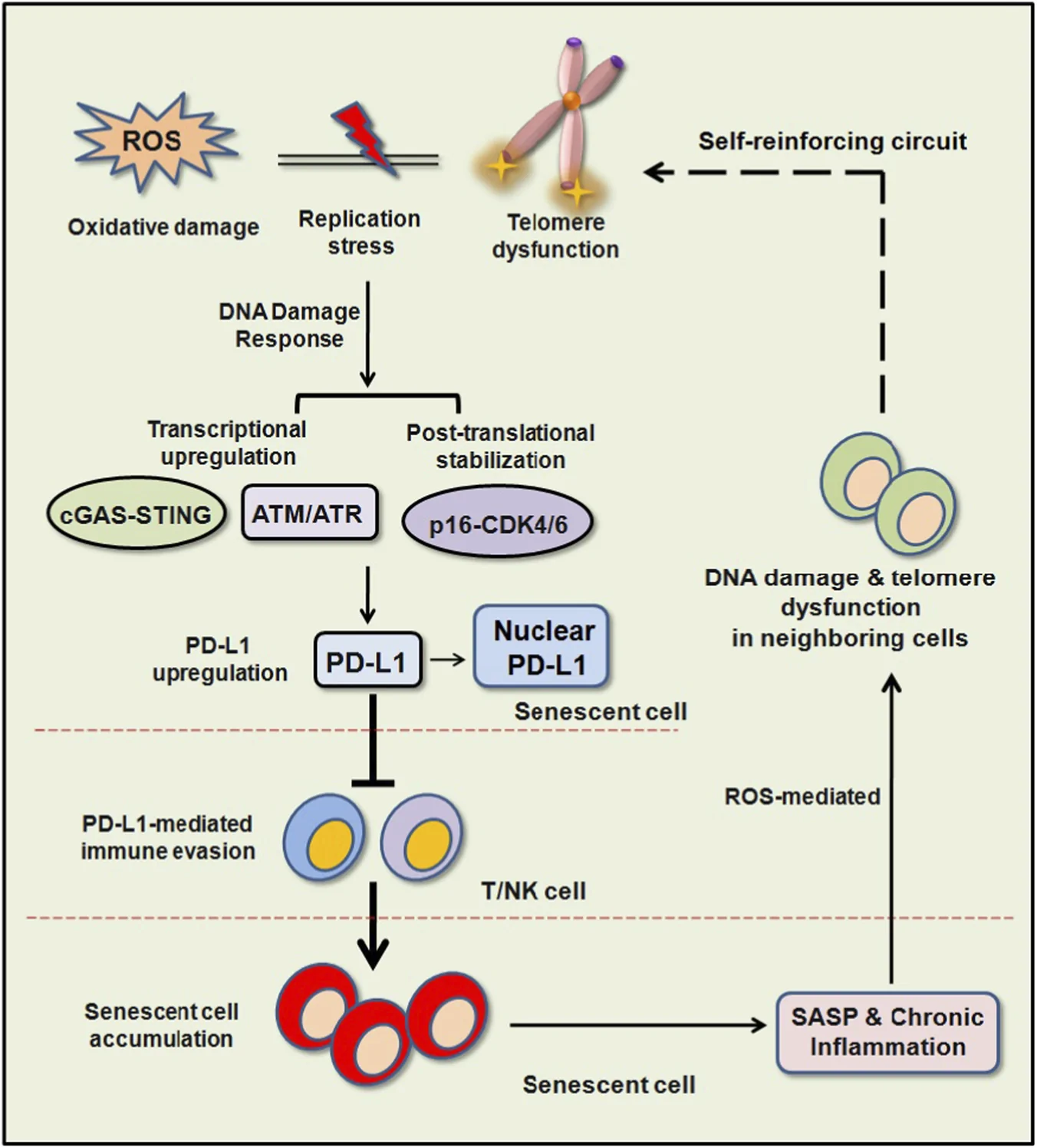
A self-reinforcing circuit linking DDR signaling to PD-L1 mediated immune evasion and senescent cell accumulation. Senescence-associated stressors, including oxidative damage, replication stress, and telomere dysfunction, activate the DDR. DDR signaling drives PD-L1 upregulation through transcriptional mechanisms (including the cGAS-STING pathway and the ATM/ATR/Chk1-STAT1/IRF1 axis, which function in a partially overlapping and interconnected manner) and post-translational stabilization via p16-mediated inhibition of CDK4/6. Elevated PD-L1 engages PD-1 on cytotoxic T cells and NK cells, enabling senescent cells to evade immune clearance. Accumulated senescent cells secrete SASP factors that promote chronic inflammation. SASP facilitates the paracrine spread of telomere dysfunction to neighboring cells, in part through neutrophil recruitment and ROS transfer, feeding back to the start of the cycle. A portion of PD-L1 also localizes to the nucleus, where it interacts with cohesin subunit SA1 to maintain telomere cohesion, a function that expands the known roles of PD-L1 beyond immune regulation. Solid arrows indicate established causal relationships; dashed arrows indicate feedback. ROS, reactive oxygen species; SASP, senescence associated secretory phenotype; SA1, stromal antigen 1; NK, natural killer.

The circuit is self-reinforcing because senescence and inflammation feed into each other. Senescent cells produce inflammatory mediators, and inflammation promotes further senescence. The DDR–PD-L1 axis is central to this loop: it protects senescent cells from immune elimination, allowing them to persist long enough to drive the inflammation that sustains the cycle. This framework also accounts for a hallmark of aging: the mutually reinforcing relationship between chronic inflammation and impaired immune function. Inflammatory signals that should activate immune responses instead reinforce PD-L1-mediated suppression. The system becomes locked in a pro-inflammatory, immunoevasive state.

Intervention could target any node in this loop, by dampening DDR signaling, blocking PD-L1, or reducing inflammation, to restore immune clearance and lower senescent cell burden (Garg et al., 2024; Majewska et al., 2024). Whether such strategies work in humans is unclear, but the circuit model offers a clear rationale for testing them.

## Future directions and open questions

The framework outlined above raises several questions that remain open.

First, the causal relationship between DDR signaling and PD-L1 upregulation *in vivo* remains to be fully defined. Although DDR signaling has been linked to PD-L1 expression in cell-based models, whether it precedes PD-L1 upregulation in aging tissues, or whether other senescence-associated signals act independently of DDR, is not yet clear. Longitudinal and tissue-specific studies will be needed to resolve this.

Second, the nuclear function of PD-L1 is only beginning to be explored. Does the nuclear pool of PD-L1 change during senescence? How does its role in telomere cohesion relate to its surface immune checkpoint function (Zhang et al., 2021)? Answering these questions may help reconcile the dual nature of PD-L1 in senescence.

Third, the relative contribution of transcriptional and post-translational mechanisms to PD-L1 upregulation likely varies across tissues. p16-mediated stabilization has been demonstrated in alveolar macrophages and epithelial cells (Majewska et al., 2024), but whether this mechanism operates with similar efficiency in other cell types remains unclear (Salminen, 2024).

Fourth, the DDR–PD-L1 axis identified here suggests potential therapeutic entry points. PD-1/PD-L1 blockade as a senolytic strategy is theoretically attractive, but systemic checkpoint inhibition carries a risk of autoimmune toxicity (Wang et al., 2018; Singh et al., 2023). Activating anti-PD-L1 antibodies that engage Fcγ receptors to eliminate PD-L1-positive senescent cells have shown preclinical promise (Majewska et al., 2024; Ovadya and Krizhanovsky, 2018; Kirkland and Tchkonia, 2017). Whether these approaches can be safely translated to humans is not yet clear.

Finally, the DDR-induced PD-L1 pathway operates in both senescence and cancer, which presents a challenge for therapeutic targeting. Interventions designed to disrupt this axis could yield benefits in both contexts (Campisi, 2013), but may also have unintended consequences depending on the cellular environment. For instance, they could affect PD-L1-expressing tumor cells or normal tissues in ways that are not fully understood. Careful context-specific evaluation will be essential before clinical translation.

## Conclusion

DDR signaling promotes PD-L1 expression in senescent cells through two routes: transcriptional induction via cGAS-STING and ATM/ATR-STAT1/IRF1, and post-translational stabilization via p16-CDK4/6. A fraction of PD-L1 also localizes to the nucleus, where it maintains telomere cohesion. These observations point to a self-reinforcing circuit. DDR-driven PD-L1 enables immune evasion, allowing senescent cells to accumulate; accumulated cells fuel inflammation, which induces DNA damage in neighboring cells and feeds back to sustain the cycle. This model explains why senescent cells persist despite their pro-inflammatory profile. It also recasts senescence as an actively maintained immunoevasive state, not a passive arrest. Therapeutically, the DDR-PD-L1 axis offers several entry points, including reducing DDR signaling, blocking PD-L1, and targeting inflammation, all of which could restore immune clearance. Whether these strategies translate into clinical benefit is uncertain, but the circuit model provides a clear rationale for testing them and a framework for understanding how senescent cells persist in aging tissues (Zhou et al., 2026; Ding et al., 2025).
